# Seasonal metabolic and growth responses of grazing beef heifers divergent in residual feed intake

**DOI:** 10.1371/journal.pone.0348184

**Published:** 2026-06-10

**Authors:** Maria Camila Londono-Mendez, Sergio Lasso-Ramirez, Carolyn Fitzsimmons, Graham Plastow, Edward W. Bork, John Basarab, Ana Caroline Cerqueira de Melo Vasco, Aghata Elins Moreira Silva, Arturo Macias Franco, Michael Vinsky, Changxi Li, Gleise Medeiros da Silva

**Affiliations:** 1 Department of Agricultural, Food and Nutritional Science, University of Alberta, Edmonton, Alberta, Canada; 2 Lacombe Research and Development Centre, Agriculture and Agri-Food Canada, Lacombe, Alberta, Canada; 3 Department of Animal and Range Sciences, Montana State University, Bozeman, Montana, United States of America; University of Illinois, UNITED STATES OF AMERICA

## Abstract

This study involved 41 crossbred, black-hided beef heifers that were measured in a drylot for residual feed intake (RFI) at 11 ± 1 months of age and classified into more (LOW-RFI; n = 21; –0.96 ± 0.70 kg DM/day) or less efficient (HIGH-RFI; n = 20; 1.40 ± 1.00 kg DM/day) groups. Heifer metabolism and growth performance were evaluated from 14 ± 1 months of age (351 ± 40 kg initial body weight [BW]) across summer and winter in Western Canada. Weather conditions were characterized using the Comprehensive Climate Index (CCI). Rumen temperature (RT) was recorded every 10 minutes using an automated bolus device. Plasma and BW were collected every 18 ± 5 days from July to August and January to March to measure urea nitrogen, non-esterified fatty acids, insulin-like growth factor 1, β-hydroxybutyric acid, leptin, free triiodothyronine (fT_3_), haptoglobin, heat shock protein 70 (HSP70), gamma-aminobutyric acid, and serotonin, and growth performance. Data were analyzed as a completely randomized design using SAS 9.4. LOW-RFI tended to have greater fT_3_ and HSP70 (*P* = 0.08), while exhibiting lower haptoglobin concentrations (*P* = 0.02) in summer. Gamma-aminobutyric acid concentrations were greater in LOW-RFI during periods of no heat stress (*P* = 0.01) and tended to decrease in HIGH-RFI under severe risk of cold stress (*P* = 0.08) based on CCI. Comparatively, HIGH-RFI had higher leptin concentrations during winter (*P* = 0.04) than LOW-RFI. During summer, HIGH-RFI exhibited greater RT between 1:00–6:00, 10:00–12:00, and 20:00–22:00 (*P* = 0.002). In contrast, HIGH-RFI had lower RTs on the coldest winter days (*P* = 0.009). In both seasons, growth performance did not differ between RFI groups (*P* ≥ 0.24). In conclusion, feed efficiency measured in the drylot was associated with subsequent metabolic responses during grazing, and these responses were influenced by weather-related stress, with more efficient animals showing greater adaptability to weather fluctuations.

## Introduction

Since the beginning of the 21st century, the frequency and intensity of extreme climate conditions have increased worldwide [1,2]. This includes a marked rise in temperatures and increased variability in extreme cold events during winter [3]. Western Canada has undergone a pronounced increase in climate variability, with the summer of 2021 notably being one of the warmest on record [4]. Climate data collected between 2007 and 2023 in Kinsella, Alberta, Canada, indicate that summer temperatures have risen by 1.4 °C, while winter temperatures have declined by 4.6 °C [5]. The highest recorded summer temperatures were 31.3 °C, 32.3 °C, and 32.7 °C during the periods 2007–2012, 2013–2018, and 2019–2023, respectively. Conversely, the lowest winter temperatures during these same intervals were –37.7 °C, –36.9 °C, and –41.6 °C [5].

The latest 2024 report on cattle populations indicates that cow–calf operations in Canada include approximately 3 million cows and replacement heifers, with around 80% of the beef sector managed under grazing conditions [6,7]. Cows in these systems may remain in the herd for up to 15 years [8] although culling commonly occurs between 4 and 6 years of age [9]. Given their extended lifespan and continuous exposure to natural environments, understanding the impacts of climate extremes is particularly critical for cow–calf producers.

Elevated climate temperatures are an external hazard for beef cattle females, posing significant risks to generate stress when heat abatement strategies are unavailable [9,10]. When body temperature increases beyond 38.5 °C, the absence of a proper equilibrium in heat dissipation and gain leads to detrimental physical responses [11] that can cause heat stress (HS). These responses include elevated respiratory and heart rates [12], increased risk of metabolic acidosis [13], abnormal behavioral patterns, impaired performance and reproductive efficiency [14], reduced nutrient intake [15], and falling immune function [16,17]. Similar detrimental responses can occur during cold stress (CS), with the primary observed responses including vasoconstriction, shivering, and elevated dry matter intake (DMI; [18]). Additionally, adaptive physiological changes may occur, including shifts in lipid, energy, and protein metabolism, which can trigger catabolism and hormonal responses [19–21]. A practical solution to address climate extremes might be genetic selection including selection on feed efficiency [22].

Genetic selection through feed efficiency has enhanced cattle breeding programs for more than 60 years [23] with residual feed intake (RFI) driving the pursuit for more feed-efficient cattle in recent years [24]. Variation in RFI is mostly explained through feed intake, digestion, metabolism, physical activity, and thermoregulation [25]. Previous research indicates cold temperatures increase the maintenance energy requirements of cattle, as more dietary energy is needed to sustain basic physiological functions under thermal stress [26]. Feed-efficient animals might cope better with these conditions, as their improved energy utilization can leave more available energy for thermoregulation [25], potentially reducing body weight (BW) loss during winter months and limiting temperature increases during summer due to a lower heat increment. Similarly, beef heifers previously classified as thermotolerant based on multiple body temperature measurements collected throughout the summer had lower RFI (more efficient) when measured in fall, indicating the potential for enhanced feed efficiency in heat-tolerant beef heifers [27]. These findings highlight the potential to explore the relationship between feed efficiency and thermotolerance, which has been limited until now [28].

Our study evaluates blood parameters, growth performance, and rumen temperature (RT) in beef heifers grazing during summer and winter in Western Canada and examined their associations with RFI. By examining the relationship between established feed efficiency and subsequent physiologic responses while grazing in varying seasonal climate conditions, this research aims to enhance animal well-being and productivity by identifying whether ongoing selection for more efficient beef heifers thrives in high-risk weather. Therefore, it was hypothesized that grazing beef heifers exhibiting higher feed efficiency are more resilient to weather-related stressors, attributed to their superior energy utilization.

## Materials and methods

### Feed efficiency test (Phase I)

This study was conducted at the Roy Berg Kinsella Research Station, University of Alberta, Kinsella, Alberta, Canada, during the summer (July–August 2022) and winter (January–March 2023) seasons. The animal protocol and procedures were approved by the University of Alberta Institutional Animal Care and Use Committee (protocol # AUP00004004). No methods of sacrifice, anesthesia, or analgesia were required in this study, as daily health checks indicated no signs of animal suffering requiring such interventions.

Heifers were sourced from a single research herd, with 91 heifers initially tested for RFI in drylot between March and May of 2022. From these, 41 black-hided heifers were selected for this study to graze during the subsequent summer and winter periods. All animals were Kinsella Composite heifers, derived from three synthetic lines (Angus–Hereford-Holstein based) developed and maintained at the Kinsella Research Ranch since 1960 [29,30].

The RFI is the difference between the actual and expected feed intake for maintenance and growth. It is a preferred measure of feed efficiency, as it is independent of BW and average daily gain (ADG). In brief, the test included a 21-day adaptation period to acclimatize cattle to the GrowSafe system (GrowSafe System, Ltd., Airdrie, Alberta, Canada) for individual daily feed intake measurements. Heifers were subsequently allocated to two pens for an 80-day RFI evaluation. All heifers were offered a single total mixed ration, consisting of barley silage and oats, provided *ad libitum* (14.6% crude protein [CP], 44% neutral detergent fiber, 32.5% acid detergent fiber, 0.97% calcium [Ca], 0.36% phosphorus [P], and 62.6% total digestible nutrients). Individual body weight (BW) was measured twice at the beginning and end of the test, while single BW measurements were obtained every 28-days. At the end of the RFI test, individual back fat (mm) was measured between the 12–13^th^ rib using an Aloka 500 V diagnostic real-time ultrasound (Aloka, Wallingford, CT) with a 17 cm 3.5 M Hz linear array transducer.

Estimates of RFI were calculated using an individual linear regression of observed BW against days on test to estimate each animal’s ADG. Initial BW and ADG were used to calculate mid-test BW and mid-test metabolic BW (MIDMBW). Linear regression of observed average daily standardized DM intake (DMI) on ADG, MIDMBW, and end-test back fat (BFEND) was used to calculate the fat-adjusted RFI (RFIf; [31]). Adjusting for fat deposition yields a feed efficiency measure that is less influenced by body composition, enabling more accurate selection for true feed conversion efficiency without negatively impacting traits like fertility [32].

Forty-one black-hided heifers (358 ± 4.78 kg BW; 14 ± 1 months of age) were selected based on coat color and classified as either more feed-efficient (LOW-RFI: RFI < 0 kg DM/d; n = 21; −0.96 ± 0.70 kg DM/d) or less feed-efficient (HIGH-RFI: RFI > 0 kg DM/d; n = 20; 1.40 ± 1.00 kg DM/d). Coat color was standardized in the selection process due to its known influence on heat load [33]. Following completion of the RFI test (Phase I), heifers were moved to a single pasture, where they grazed under a rotational grazing system during Phase II. In Phase III, unrolling hay bales were offered daily at 9:00.

### Genomic breeding composition and retained heterozygosity

Genetic breed composition and hybrid vigor were evaluated within groups to determine whether genetic variation influences RFI and physiological responses. Genotyping was performed on ear biopsies (TypiFix^TM^, Agrobiogen GmbH, Hilgertshausen, Germany) at weaning. Biopsies were sent to Delta Genomics (Edmonton, AB, Canada) for DNA analysis. Then, the genomic breed composition fraction (gBC) was predicted using Admixture software [34] based on 15,401 common SNPs between the animal and a reference panel of 14 pure cattle breeds, including 4,721 individuals from Black Angus, Red Angus, Charolais, Simmental, Hereford, Limousin, Gelbvieh, Salers, Maine Anjou, Shorthorn, Holstein, Brown Swiss, Jersey, and Galloway. The gRHET was calculated using a modified formula: $\text{gRHET}=\text{1}\;-\;[\sum (Pi^{\text{2}})]\;/\;n$, where Pi represents the fraction of each breed—specifically, Black and Red Angus combined—based on a previously described method [35].

### Sampling periods (Phases II and III)

In Phase II, climate data, plasma samples, RT, BW, and rump (Gluteus Medius) and rib fat measurements were collected during the summer (July to August 2022) on days 0, 14, 28, and 42 of the study. Subsequently, in winter (January to March 2023), grazing occurred within Phase III that included a second round of sampling on days 185, 204, 227, and 239, relative to the start of summer sampling. The fall season was excluded due to the absence of expected extreme climate conditions. In Phases II and III, heifers were treated as a single herd, allowing all animals equal access to the same environment.

During summer, heifers grazed a pasture predominantly composed of *Poa pratensis, Bromus inermis Leyss*, and *Hesperostipa curtiseta* at 2.72 animal unit month/ha over 7 weeks and were exposed to two breeding bulls for natural service. In winter, heifers were offered free-choice of *Medicago sativa* hay twice daily at the same location. Hand-plucked forage and hay samples were collected on days 0, 14, 28, 42, 185, 204, 227, and 239 for chemical composition analysis, which was performed by a commercial laboratory (Down to Earth Labs, Lethbridge, Canada; Table 1). Herbage mass (DM/kg) was estimated on days 0, 14, 28, and 42, using a randomized sampling approach within each pasture, stratified according to topographical variations. A quadrat (0.25 m^2^) was positioned over a randomly chosen sample area, ensuring that only biomass rooted within this quadrat was clipped 2 cm above ground. Following this, samples underwent drying at a temperature of 79 °C for one week and were immediately weighed after extraction from dryers.

**Table 1:** Herbage mass and chemical composition of pasture and hay consumed by beef heifers with divergent residual feed intake (RFI) during summer (July to August of 2022) and winter (January to March of 2023).

	Summer days (pasture)				Winter days (hay)			
*Item* ^1^	0	14	28	42	185	204	227	239
HM, kg/ha	1374	1000	996	1793	–	–	–	–
HA, kg DM/ kg BW	1.80	1.25	1.17	2.03	–	–	–	–
			%					
CP	12.0	22.3	16.1	9.1	14.3	7.9	8.1	8.7
ADF	30.9	25.8	27.9	33.3	29.5	45.1	31.8	45.3
NDF	58.2	46.9	55.4	59.5	39.5	58.2	50.1	54.0
TDN	62.8	65.1	64.1	61.7	59.5	50.6	57.7	49.0
			ppm					
Copper	3.4	7.6	3.8	1.9	6.4	5.3	3.2	4.7
Manganese	45.1	44.6	35.6	41.9	61.9	29.9	10.8	10.3
Zinc	21.3	29.8	24.4	16.7	22.4	17.2	11.5	9.7
Iron	76.4	92.7	87.6	101.1	75.8	55.1	14.8	15.6
			%					
Sulfur	0.1	0.2	0.2	0.2	0.2	0.1	0.1	0.1
Sodium	BDL^2^	BDL^2^	BDL^2^	BDL^2^	0.1	0.1	0.1	0.1
Potassium	1.7	3.0	1.7	1.4	2.5	1.3	1.8	1.6
Phosphorous	0.3	0.4	0.3	0.3	0.2	0.1	0.1	0.1
Calcium	0.3	0.3	0.3	0.4	0.9	0.8	0.5	0.9
Magnesium	0.1	0.2	0.2	0.2	0.2	0.2	0.2	0.2

^1^HM: Herbage mass, HA: Herbage allowance, DM: dry matter, BW: body weight, CP: Crude protein, ADF: Acid detergent fiber, NDF: Neutral detergent fiber and, TDN: Total digestible nutrients.

^2^BDL: Below the detection limit.

### Weather data

The comprehensive climate index (CCI) [36] was used as the indicator of environmental stress for grazing beef cattle, using data from a weather station within 1 km where the heifers were placed. The CCI was calculated according to Mader et al. [36] as the sum of air temperature (Ta), relative humidity (RH; $RH_{correctionfactor}=\;e^{\left(\text{0}.\text{0}\text{0}\text{1}\text{8}\text{2}\times RH+\text{1}.\text{8}\times {\text{1}\text{0}}^{-\text{5}}\times Ta\times RH\right)}\times \left(\text{0}.\text{000054}\times {\text{Ta}}^{\text{2}}+\text{Ta}-\text{0}.\text{0246}\right)\times (\text{RH}-\text{30})$, wind speed (WS; ${\text{WS}}_{\text{correctionfactor}}=-\frac{\text{6}.\text{5}\text{6}}{e^{{\left(\frac{\text{1}}{\text{2}.\text{2}\text{6}\times WS+\text{0}.\text{2}\text{3}}\right)}^{\text{0}.\text{4}\text{5}}}\times {\left(\text{2}.\text{9}+\text{1}.\text{1}\text{4}\times {\text{1}\text{0}}^{-\text{6}}\times WS^{\text{2}.\text{5}}-\right)}^{-\text{2}}}$), and solar radiation (RAD; $RAD_{correctionfactor}=\text{0}.\text{0}\text{0}\text{7}\text{6}\times RAD-\text{0}.\text{0}\text{0}\text{0}\text{0}\text{2}\times RAD\times Ta+\text{0}.\text{0}\text{0}\text{0}\text{0}\text{5}\times Ta^{\text{2}}\times \sqrt{RAD}+\text{0}.\text{1}\times Ta-\text{2}$).

For each of the summer and winter seasons, the calculated CCI categorized the weather conditions into one of the following thresholds: non-stress, mild, moderate, severe, extreme, and extreme danger conditions (< 25, 25–30, > 30–35, > 35–40, > 40–45 and, > 45 vs. > 0, 0 to −10, < −10 to −20, < −20 to −30, < −30 to −40 and, < −40, respectively for each season [36]). The mean, minimum, and maximum of the climate variables between each sampling period (days 0–14, 14–28, 28–42, 185–204, 204–227, and 227–239) and CCIs are reported in Table 2.

**Table 2:** Weather descriptive statistics with average, maximum (Max) and minimum (Min) values for each variable during the summer and winter seasons (July to August 2022 and January to March 2023; respectively).

	Day of the experiment relative to the start of the summer sampling					
Climate variable^1^	0-14	14-28	28-42	185-204	204-227	227-239
Air temperature (°C)	18.3	17.2	19.1	−8.2	−9.0	−14.7
Max	29.6	28.4	31.1	4.5	5.7	3.0
Min	7.2	5.8	6.0	−25.6	−32.6	−34.1
Relative humidity (%)	74.3	79.5	72.9	88.7	77.2	77.7
Max	100	100	100	100	98.1	94.9
Min	34.8	35.1	27.1	66.7	48.2	49.8
Solar radiation (w/m^2^)	297.6	256.1	265.8	50.7	88.5	135.6
Max	905.1	853.1	838.1	367.4	492.8	604.5
Min	0.2	0.3	0.2	0	0	0
Wind speed (km/h)	8.9	9.8	7.1	9.4	10.0	10.9
Max	22.1	28.7	16.8	37.0	35.8	22.4
Min	1.6	1.6	1.2	0.3	1.6	1.7
CCI ^2^	25	24	26	−14	−14	−19
CCI Mean Index	Mild	Mild	Mild	Moderate	Moderate	Moderate

^1^Values from meteorological station placed within approximately 1 km from the experimental site. ^2^CCI = Comprehensive Climate Index as described in the methods [36].

### Growth performance

Body weights were obtained using calibrated scales with radio frequency identification readers (Gallagher Smart TSi, Gallagher Australia Pty, Ltd.). In summer, the initial BW was calculated as the average of full BW on d −1 and 0, and on d 184 and 185 for the winter, while the final BW was the average of day 42 and 43 for the summer, and the single reading on d 238 for winter. Additionally, BW was intermittently collected every 18 ± 5 days for a total of 42 days during summer and 18 ± 8 days for 54 days during winter to assess ADG between sampling days. Rib and rump fat thickness were estimated using an Aloka 500 V diagnostic real-time ultrasound (Aloka, Wallingford, CT) with a 17 cm, 3.5 M Hz linear array transducer at the beginning and the end of each sampling period (Phase II and III).

### Blood parameters and rumen temperature

Blood samples were collected through jugular venipuncture during the summer and winter seasons (days 0, 14, 28, and 42 for summer, and days 185, 204, 227, and 239 for winter) into sodium heparin tubes (Vacutainer, Becton Dickinson, Franklin Lakes, NJ) and placed on ice until centrifuged at 3200 rpm for 15 minutes at 4 °C. Plasma was then transferred to polypropylene vials (12 × 75 mm; Fisherbrand; Thermo Fisher Scientific Inc., Waltham, MA) and stored at −80 °C until further analysis.

Commercial ELISA kits were used to determine free triiodothyronine (fT_3_; inter- and intra-assay coefficients of variation were 5.37 and 5.70, respectively; Cat. No. CSB-EQF027510BO Cusabio Technology llc, Houston, TX, USA), heat shock protein 70 (HSP70; inter- and intra-assay coefficients of variation were 6.76 and 6.01, respectively; Cat. No. CSB-E13452B Cusabio Technology llc, Houston, TX, USA), bovine β-Hydroxybutyric acid (BHBA; inter- and intra-assay coefficients of variation were 7.38 and 7.91, respectively; Cat. No. CSB-E10056b Cusabio Technology llc, Houston, TX, USA), blood urea nitrogen (BUN; inter- and intra-assay coefficients of variation were 3.93 and 2.20, respectively; cat. No. EIABUN Invitrogen, Carlsbad, CA, USA), haptoglobin (Hp; inter- and intra-assay coefficients of variation were 4.05 and 4.34, respectively; Cat. No. E-10HPT ICL, Newberg, OR, USA), gamma-aminobutyric acid (GABA; inter- and intra-assay coefficients of variation were 8.55 and 5.79, respectively; Cat. No. BOEB1223 Assay Genie, Dublin, Leinster, IRL), serotonin (5-HT; inter- and intra-assay coefficients of variation were 9.43 and 6.55, respectively; Cat. No. BOEB1217 Assay Genie, Dublin, Leinster, IRL), insulin-like growth factor type-1 (IGF-1; inter- and intra-assay coefficients of variation were 7.15 and 9.61, respectively; Cat. No. SG100B R&D Systems, Minneapolis, MN, USA), non-esterified fatty acids (NEFA; inter- and intra-assay coefficients of variation were 2.08 and 5.30, respectively; Cat. No. 999–34691, 995–34791, 991–34891, 993–35191, 276–76491 Fujifilm Wako Diagnostics, Mountain View, CA, USA), and leptin (LEP; inter- and intra-assay coefficients of variation were 9.46 and 9.74, respectively; Cat. No. EK760144, AFG bioscience, Northbrook, IL, USA) concentrations in plasma.

On day 14, an automated RT logger (Smart Rumen Bolus, Moonsyst, Hungary) was orally administered using a bolus gun to each animal, to record individual RT at 10-minute intervals. For RT, the summer season was defined as days 14–42, and the winter season as days 185–239.

### Statistical analyses

Except for BW, ADG data were analyzed as a completely randomized design with repeated measurements using the GLIMMIX procedure of SAS (version 9.4; SAS Institute Inc., Cary, NC, USA). The gBC and gRHET were analyzed using t-tests to assess potential differences in mean values among the breeds that make up the Kinsella composite population and hybrid vigor effect.

Data on RT were summarized and analyzed by hour (0–24 h) and by day of the study. Individual heifers were the experimental unit for all analyses and included as a random effect nested within treatment, and RFI classification (LOW vs. HIGH) obtained in Phase I was used as the fixed effect for all analyses (i.e., BW, ADG, and fat thickness). For RT and plasma measurements, data were analyzed as repeated measures and tested for fixed effects of RFI, day, and RFI × day interaction (or hour for RT only). For RT by the hour, day of study was included as a random effect. Decreases in RT associated with water or snow intake (i.e., values below 36.5 °C) were manually removed from the raw data for each season before analysis. A simple linear regression model was used to evaluate the effect of gRHET on RFI. The gRHET was used as a fixed covariate in the model to estimate the effects of animal heterosis. However, it was not significant (*P* = 0.39) and therefore removed from the final statistical model.

Residuals and variables were tested for normality using the Kolmogorov-Smirnov test (*P* > 0.05), while non-normal data (*P* < 0.05) were power-transformed to normality using the box-cox transformations. All blood variables except for BUN in the summer season were transformed. Means were back-transformed for data reporting and compared through the Tukey-Kramer test. The lowest Akaike Information Criterion was used to select the optimal covariance structures for repeated measures. Significance was set at *P* < 0.05, and tendencies were declared when *P* ≥ 0.05 to ≤ 0.10.

## Results and discussion

**Fig 1:**
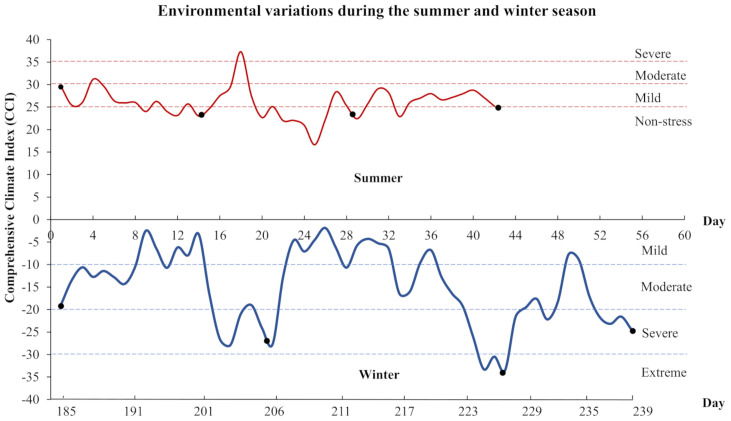
Climate conditions on individual sampling days as classified according to the Comprehensive Climate Index (CCI) [36]. During the summer sampling period (July to August 2022), conditions ranged from mild heat stress to non-stress, with day 0 classified as mild, while day 14, 28, and 42 as non-stress (●). In contrast, winter sampling (January to March 2023) indicated moderate to extreme cold stress, with days 185 classified as moderate, days 204 and 239 classified as severe, and day 227 as extreme (●).

During this study, cattle were exposed to a range of weather conditions, from no stress to severe risk of HS. During winter, heifers experienced conditions ranging from no risk of CS to an extreme level of CS (Fig 1). Both seasons represented significant climate challenges for beef cattle raised outdoors. In particular, summer temperatures at the study site (Kinsella, AB, Canada) have increased by 1.4 °C, while winter temperatures have dropped by 4.6 °C over the past 16 years, highlighting the region’s shifting and extreme climatic conditions [5]. Currently, equations are available to estimate the effects of climate fluctuations on cattle stress [33,36–38]. One of the most recently introduced metrics is the CCI, an alternative indicator of thermal stress in grazing cattle [36] that incorporates RAD and WS. Adjusting for those variables is crucial because RAD and WS significantly influence how animals dissipate or retain heat [36]. Based on the averaged CCI, this research found that the summer of 2022 posed lower risks of stress (mild to moderate) compared to the winter season (moderate; Table 2). Nonetheless, the climate extremes observed during the experiment were found to affect the ability of cattle to maintain stable body temperature and support normal physiological functions [39].

Although continuous monitoring of body temperature can be challenging, several technologies have been developed to enhance the ability to track animal body temperature more effectively [40,41]. For ruminants, RT can be used as a proxy for body temperature, with a moderate to strong correlation [42]. Under HS conditions, increases in body temperature and RT can impact cattle daily routines and negatively affect feeding behavior and nutrient intake. Adaptative behaviours include shifting activities, such as grazing, to cooler times of the day, like early morning or late evening [43]. Heat stress is known to reduce blood flow to the gastrointestinal tract, which impairs nutrient absorption and disrupts appetite regulation [44]. Additionally, heifers with elevated body temperatures are likely to divert more feed energy toward thermoregulation rather than productivity, ultimately decreasing overall production efficiency [43]. Previous research has shown that less feed-efficient steers tend to produce more heat, which may be attributed to differences in metabolic efficiency [45]. Conversely, during the winter, body heat loss to the environment can lower core temperature and trigger the opposite feeding response, an increase in feed intake, to support thermogenesis [46,47].

**Fig 2:**
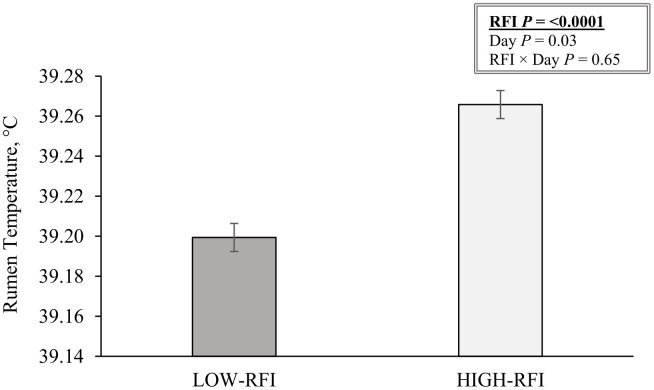
Rumen temperature (RT) of crossbred beef heifers previously classified as more (LOW-RFI) or less (HIGH-RFI) feed efficient. LOW-RFI heifers exhibited lower RT than HIGH-RFI during the summer season (*P* < 0.001; 39.2 vs. 39.3; SEM ± 0.007 °C). SEM: Standard error of the mean. RFI: Residual feed intake. HIGH-RFI: Less efficient individual tested for residual feed intake with a positive DMI. LOW-RFI: More efficient individual tested for residual feed intake with a negative DMI.

**Fig 3:**
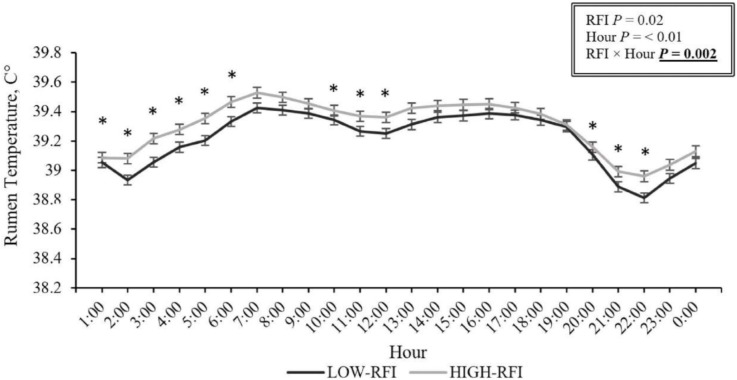
Effect of RFI × hour interaction on rumen temperature (RT) of crossbred beef heifers previously classified as more (LOW-RFI) or less (HIGH-RFI) feed efficient. HIGH-RFI heifers had greater rumen temperatures from 1:00 to 6:00, 10:00 to 12:00 and 20:00 to 22:00 compared with LOW-RFI animals in the summer (*P* = 0.002; SEM ± 0.028 °C). Data were recorded every 10 min and averaged by hour. *Within hour, means with an asterisk are different (*P* < 0.05). SEM: Standard error of the mean. RFI: Residual feed intake. HIGH-RFI: Less efficient individual tested for residual feed intake with a positive DMI. LOW-RFI: More efficient individual tested for residual feed intake with a negative DMI.

During the summer grazing season, an effect of RFI as previously measured in the drylot, on RT was observed (*P* < 0.001), with HIGH-RFI (i.e., less feed efficient) heifers exhibited higher RT compared to LOW-RFI heifers when analyzed across days (Fig 2). Also, an RFI × hour interaction was detected for RT during summer (*P* = 0.002), whereby HIGH-RFI heifers had greater RT from 1:00–6:00, 10:00–12:00 and 20:00–22:00 (Fig 3). This finding aligns with previous research [48] reporting that beef cattle with greater feed efficiency, based on lower RFI, demonstrated superior thermoregulation, as measured by infrared thermography. Similarly, a study in swine found that animals with higher feed efficiency had enhanced thermoregulatory capacity [49]. In poultry, heat production has been identified as a potential physiological mechanism contributing to variation in feed efficiency [50], a relationship that has also been well-documented in beef cattle [51]. The reduced thermoregulatory ability of HIGH-RFI heifers may be attributed to their expected higher forage intake, which increases heat increment and is typically associated with lower metabolic feed efficiency, reflecting suboptimal utilization of adenosine triphosphate [52]. Greater forage consumption increases the heat increment of feeding, potentially elevating body temperature and thereby making it more difficult for cattle to cope with hot climate conditions [51]. While it is important to note that actual forage intake was not measured in this study, and our RFI estimates were performed in drylot during the spring, it does highlight the sensitivity of RFI metrics and their ability to carry over between seasons within the same animals.

**Fig 4:**
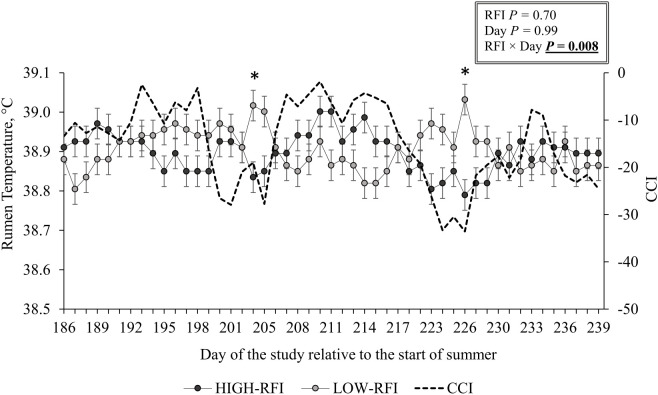
Effect of RFI × day interaction on rumen temperature (RT) of crossbred beef heifers previously classified as more (LOW-RFI) or less (HIGH-RFI) feed efficient. HIGH-RFI heifers exhibited lower rumen temperatures on days 203 and 226, when climate conditions posed severe and extreme levels of cold stress, as classified by the Comprehensive Climate Index (CCI; *P* = 0.0086; SEM ± 0.038 °C). *Within day, means with an asterisk are different (*P* < 0.05). SEM: Standard error of the mean. RFI: Residual feed intake. HIGH-RFI: Less efficient individual tested for residual feed intake with a positive DMI. LOW-RFI: More efficient individual tested for residual feed intake with a negative DMI. CCI: Comprehensive Climate Index [36].

During the winter season, a significant RFI × day interaction was observed (*P* = 0.0086; Fig 4), with LOW-RFI heifers exhibiting higher RT than the HIGH-RFI group on days 203 and 226. These days notably coincided with climate conditions classified by the CCI as posing severe to extreme risk of CS, respectively. In non-ruminants, it has been reported that less feed-efficient newborn piglets had more difficulty maintaining body temperature, as evidenced by lower ear tip temperatures, compared to more feed-efficient cohorts [53]. This suggests that more efficient piglets can better adjust their body temperature during stressful conditions. In a similar outcome, our results demonstrate that LOW-RFI heifers exhibited superior thermoregulation abilities, both by maintaining higher RT during extreme cold winter days, as well as lower temperatures during hot summer days. These responses may reflect differences in thermoregulatory capacity between RFI groups [27]. Furthermore, during summer, the expected lower feed intake of LOW-RFI heifers may have reduced the heat increment of feeding and limited heat accumulation. In contrast, the lower RT observed in HIGH-RFI animals during winter may indicate greater susceptibility to CS, as severe CS can reduce feed intake, rumen fermentation activity, and body temperature. However, feed intake was not measured during the summer and winter periods, and further studies are needed to confirm this mechanism. This finding highlights the enhanced capacity to cope with strong seasonal variation in climate conditions for LOW-RFI heifers, which are particularly pronounced in temperate environments such as those in western Canada.

**Fig 5:**
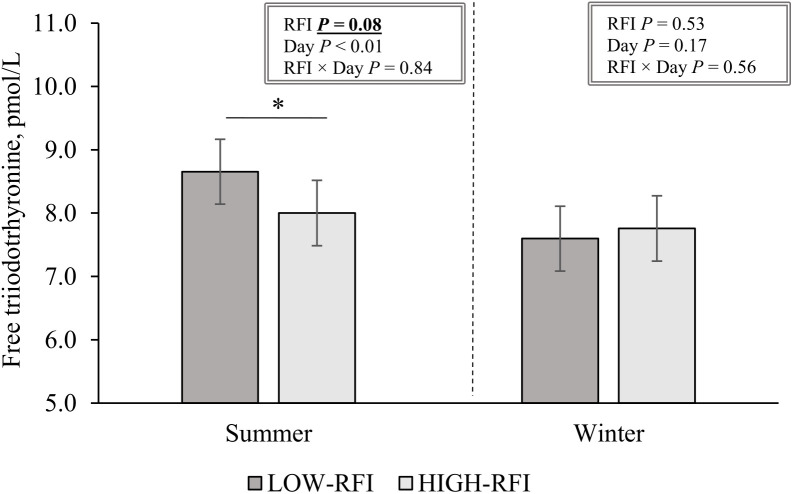
Plasma concentrations of free triiodothyronine (fT_3_) measured during the summer and winter (summer 2022 and winter 2023) in crossbred beef heifers previously classified as more (LOW-RFI) or less (HIGH-RFI) feed efficient. During the summer, there was a tendency for an RFI effect, with the LOW-RFI group exhibiting higher fT_3_ concentrations compared to the HIGH-RFI group (*P* = 0.08; 8.65 vs. 8.00; SEM ± 1.032 pmol/L). In contrast, no significant differences were observed between groups during the winter season (*P* = 0.53). *Within RFI, means with an asterisk tended to be different (*P* = 0.08). SEM: Standard error of the mean. RFI: Residual feed intake. HIGH-RFI: Less efficient individual tested for residual feed intake with a positive DMI. LOW-RFI: More efficient individual tested for residual feed intake with a negative DMI.

Supporting the RT data, LOW-RFI heifers tended to have higher concentrations of fT_3_ during summer compared to HIGH-RFI heifers (*P* = 0.08; Fig 5). These results further suggest that more feed-efficient heifers were less affected by HS, as their metabolic heat production and hormonal downregulation of fT_3_ typically used to reduce heat production was not required. The HS in cattle induces several physiological adaptations, including a reduction in circulating thyroid hormone concentrations [54]. This decrease reflects an adaptive response to lower metabolic heat production and facilitates coping with elevated climate temperatures [55]. Additionally, thyroid hormones are positively correlated to weight gain and increased basal metabolic rate, whereas lower concentrations can be found in dairy cows exposed to HS [56]. Under thermal stress conditions, Limousin cattle exhibited a decrease in triiodothyronine hormone concentration to 76% of the baseline levels observed under thermoneutral conditions. This reduction serves as a compensatory response to achieve lower metabolic heat production [57].

The benefits of understanding whether elevated circulating levels of free fT_3,_ confer physiological advantages by entering cells, translocating to the nucleus, and binding to receptor complexes at DNA level, thus regulating gene expression through type 1 and type 2 signaling pathways [58] and regulating mitochondrial gene [59]. Those capabilities are crucial for several physiological functions, such as basic maintenance, muscle growth, and the onset of early puberty in heifers [51–57,60,61]. Concentration of fT_3_ is also affected by stress due to the negative feedback effect of cortisol on the hypothalamic-pituitary-adrenal (HPA) axis [62]. This sensitivity leads to significant reductions in fT_3_ concentrations during stress exposure, as observed in Limousin bulls during prolonged transport [63]. High cortisol levels can impair HPA axis function, as corticosteroids reduce the activity of the 5-deiodinase enzyme, which converts thyroxine to fT_3_ [64]. Heifers exposed to HS typically experience reduced feed intake, which may be accompanied by decreased thyroid secretion [57]. However, future research should validate these findings in extensive pasture-based feeding systems, as feed intake was not estimated during phases II and III of our trial. Additionally, no differences in fT_3_ were observed during the winter season (*P* = 0.53; Fig 5).

In the present study, an effect of day was observed for NEFA and BHBA concentrations during both summer and winter (*P* < 0.001; Table 3), whereas no effect of RFI or RFI × day interaction was detected (*P* ≥ 0.71; Table 3). Heat stress has been shown to affect circulating concentrations of NEFA and BHBA [65,66], typically associated with reduced feed intake and increased mobilization of lipid reserves [67], which can occur under stressful conditions. However, heat-stressed cattle, despite reduced feed intake, often exhibit elevated insulin concentrations and altered energy metabolism, which may contribute to reduced lipid mobilization [68]. This may reflect a strategy to limit metabolic heat production, as β-oxidation of NEFA generates more heat than carbohydrate oxidation [69]. In agreement with previous studies reporting limited changes in NEFA during heat stress [69,70], our results suggest that NEFA concentrations remained relatively stable during weather conditions that might induce stress (Fig 1 and Table 3).

**Table 3:** Blood parameters measured during summer and winter days of beef heifers previously classified as more (LOW) or less (HIGH) feed efficient based on residual feed intake (RFI).

Item^1^	RFI		Day of the experiment relative to the start of the summer sampling^3^								SEM^2^	*P-*value		
	LOW	HIGH	0	14	28	42	185	204	227	239		RFI	Day	RFI × Day
*Summer*														
fT_3,_ pmol/L	8.65	8.00	6.9^b^	7.2^b^	9.1^a^	10.6^a^	–	–	–	–	1.040	0.087	< 0.001	0.840
IGF-1, ng/ml	10.26	9.30	11.2^a^	11.7^a^	9.8^a^	7.0^b^	–	–	–	–	1.088	0.258	< 0.001	0.902
BHBA, nmol/L	233.6	219.4	237.8^ba^	199.3^c^	218.5^bc^	253.7^a^	–	–	–	–	1.040	0.256	< 0.001	0.318
NEFA, mEq/L	0.220	0.191	0.279^a^	0.150^c^	0.213^b^	0.205^b^	–	–	–	–	0.004	0.124	< 0.001	0.719
BUN, mg/dL	37.2	34.7	21.2^b^	38.0^a^	38.2^a^	46.5^a^	–	–	–	–	0.023	0.404	< 0.001	0.081
LEP, ug/L	7.9	7.7	4.1^c^	8.4^b^	13.4^a^	8.0^b^	–	–	–	–	1.075	0.732	< 0.001	0.490
HSP70, ng/ml	3.2	2.99	3.2^b^	3.0^ba^	2.9^c^	3.3^a^	–	–	–	–	0.857	0.101	< 0.001	0.384
Hp, ng/ml	1275	2185	1939^ba^	1029^b^	2760^a^	1408^b^	–	–	–	–	1.222	0.020	< 0.001	0.852
GABA, ng/ml	8.1	8.2	7.7	7.9	8.7	8.5	–	–	–	–	0.078	0.825	0.201	0.011
5-HT, ng/ml	48.7	38.2	25.4^b^	49.6^a^	42.6^a^	59.6^a^	–	–	–	–	0.185	0.141	< 0.001	0.345
*Winter*														
fT_3,_ pmol/L	7.60	7.76	–	–	–	–	8.00	7.22	7.75	7.74	1.025	0.536	0.179	0.562
IGF-1, ng/ml	29.3	28.8	–	–	–	–	38.7^a^	33.8^a^	26.1^b^	19.6^c^	0.040	0.829	< 0.001	0.564
BHBA, nmol/L	217.45	233.91	–	–	–	–	269.0^a^	272.1^a^	128.2^b^	233.4^ba^	30.277	0.673	< 0.001	0.726
NEFA, mEq/L	0.448	0.426	–	–	–	–	0.230^c^	0.453^b^	0.619^a^	0.497^ba^	0.001	0.454	< 0.001	0.797
BUN, mg/dL	7.1	7.8	–	–	–	–	7.0^b^	16.8^a^	4.8^b^	6.6^b^	0.001	0.602	< 0.001	0.405
LEP, ug/L	4.5	5.2	–	–	–	–	3.7^b^	7.5^a^	4.7^b^	4.6^b^	0.025	0.048	< 0.001	0.581
HSP70, ng/ml	6.5	6.3	–	–	–	–	6.1^b^	6.4^b^	7.2 ^a^	6.2^b^	0.047	0.599	0.037	0.500
Hp, ng/ml	724	707	–	–	–	–	834^a^	591^b^	989^a^	539^b^	1.086	0.782	< 0.001	0.057
GABA, ng/ml	5.9	5.5	–	–	–	–	7.1^a^	7.2^a^	4.4^b^	4.7^b^	1.082	0.474	< 0.001	0.088
5-HT, ng/ml	36.0	34.3	–	–	–	–	56.7^a^	22.8^b^	31.9^b^	37.1^ba^	1.167	0.752	< 0.001	0.393

^1^fT_3_: Free triiodothyronine, IGF-1: Insulin-like growth factor type 1, BHBA: Bovine β-Hydroxybutyric acid, NEFA: Non-esterified fatty acids, LEP: Leptin, HSP70: Heat shock protein 70, Hp: Haptoglobin, 5-HT: Serotonin, BUN: Blood urea nitrogen and, GABA: Gamma-aminobutyric acid.

^2^SEM: Standard error of the mean.

^3^Within a row, means with different letter superscripts differ, *P* < 0.05.

During winter, NEFA concentrations were highest on the day classified as posing extreme cold stress risk by the CCI, with a significant increase observed on day 227 (*P* < 0.001; Fig 1), likely reflecting a transient negative energy balance due to increased energy demands under severe cold conditions, as previously reported in cattle exposed to CS [70]. Furthermore, the lack of differences in BHBA concentrations among RFI groups during winter aligns with previously reported findings [71].

**Fig 6:**
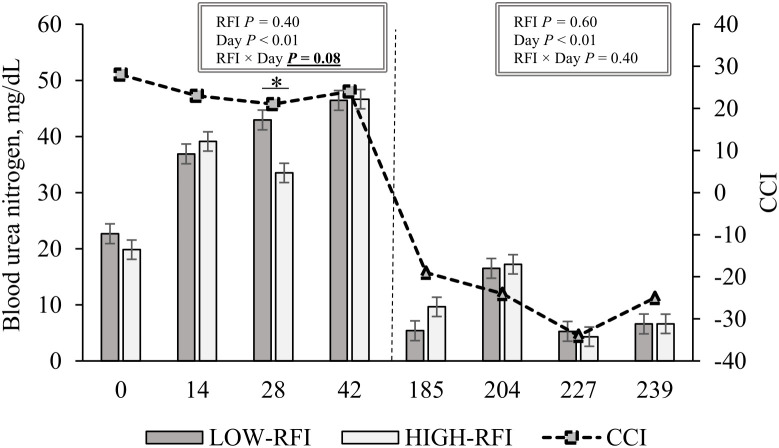
Plasma concentrations of urea nitrogen (BUN) measured during the summer and winter (summer 2022 and winter 2023) in crossbred beef heifers previously classified as more (LOW-RFI) or less (HIGH-RFI) feed efficient. A tendency for an RFI × day interaction was observed during the summer, with LOW-RFI heifers showing higher BUN concentrations compared to HIGH-RFI heifers on day 28 (*P* = 0.08; 43 vs. 34; SEM ± 3.52 mg/dL). No significant RFI × day interaction was detected during the winter season (*P* = 0.40). *Within RFI, means with an asterisk tended to be different (*P* = 0.08). SEM: Standard error of the mean. RFI: Residual feed intake. HIGH-RFI: Less efficient individual tested for residual feed intake with a positive DMI. LOW-RFI: More efficient individual tested for residual feed intake with a negative DMI. CCI: Comprehensive Climate Index [36].

Blood urea nitrogen is associated with protein metabolism and the efficiency of amino acid utilization, reflecting the degree of nitrogen utilization in the rumen [72], and has been reported to increase 4 hours after cattle are fed [73]. A tendency for an RFI × day interaction [*P* = 0.08; Fig 6] in the summer season was observed for BUN. LOW-RFI heifers had greater BUN than HIGH-RFI heifers (43 vs. 34 mg/dL, respectively) when exposed to a non-stress climate risk of generating HS (day 28). A potential explanation for this could be the greater abundance of the *Prevotella* genus in the rumen of more feed-efficient animals, which may enhance nitrogen metabolism [74]. This improvement in microbial activity could lead to more efficient protein metabolism, and consequently, higher BUN concentrations [72,74]. Moreover, while BUN concentrations were elevated in both LOW-RFI and HIGH-RFI groups compared to a previous report [75], it is important to note that heifers in this study had access to feed before blood sampling, which was conducted early in the morning while they were on pasture. Free choice feed access, along with the higher CP content in their diet at that time of year (Table 1), may explain the elevated BUN levels observed in our study. On the other hand, high BUN levels in heat-stressed cattle indicate metabolic shifts, which can be partly explained by changes in microbial fermentation that reduce the use of rumen ammonia for microbial crude protein synthesis [76]. High levels of BUN can result from the ineffective assimilation of rumen ammonia into microbial protein and the liver’s process of deaminating amino acids released from skeletal muscle [74,77]. Future research should investigate nitrogen metabolism in more feed-efficient cattle in response to climate extremes.

Reduced BUN concentrations are typically observed when cattle decrease feed intake to mitigate additional heat production associated with digestion [78]. Accelerated protein catabolism in the muscles provides more energy substrate for thermoregulation [78], which may result in lower growth performance in heifers [79]. In this study, however, no significant differences were observed in growth performance between cattle categorized as LOW-RFI and HIGH-RFI during either summer or winter (*P* ≥ 0.24; Table 4). This lack of difference may be explained by the disappearance of fat deposition differences after adjusting RFI for backfat, suggesting that feed efficiency primarily reflects intrinsic metabolic variation rather than performance between feed efficiency groups [32].

**Table 4:** Growth performance during the summer and winter seasons of beef heifers previously classified as more (LOW) or less (HIGH) feed efficient based on residual feed intake (RFI).

	---------- RFI ----------			
Item^1^	LOW	HIGH	SEM^*3*^	*P*-value^4^
n	21	20	–	–
RFI Classification	−0.96	1.40	0.193	< 0.001
*RFI test*				
BW, kg				
Initial	230	235	3.57	3.45
Final	376	377	4.77	0.85
*Summer*				
ADG, kg				
0 to 14 day	0.75	0.93	0.105	0.25
14 to 28 day	1.01	1.07	0.270	0.88
28 to 42 day	0.80	0.64	0.287	0.71
0 to 42 day	0.35	0.37	0.030	0.70
*BW, kg*				
Initial	359	357	4.71	0.83
Final	394	394	4.86	0.95
*Fat deposition, mm*				
Initial Rib	2.87	2.57	0.196	0.28
Final Rib	2.39	2.48	0.244	0.80
Initial Rump	4.65	4.42	0.347	0.64
Final Rump	3.87	3.76	0.317	0.81
*Winter*				
ADG, kg^2^				
185 to 204 day	0.5	0.1	0.278	0.37
204 to 227 day	−0.4	−0.2	0.097	0.24
227 to 239 day	2.3	2.4	0.234	0.86
185 to 239 day	0.5	0.5	0.095	0.75
BW, kg^2^				
Initial	433	440	7.62	0.56
Final	462	466	6.98	0.69
*Fat deposition, mm*				
Initial Rib	3.1	2.8	0.234	0.31
Final Rib	–	–	–	–
Initial Rump	5.3	4.8	0.365	0.40
Final Rump	–	–	–	–

^1^RFI = Residual Feed Intake, BW = body weight, ADG = average daily gain.

^2^Pregnancy weight was corrected as described in the methods [80].

^3^SEM = Standard error of the mean.

^4^Statistical significances at *P* < 0.05 and tendency between *P* ≥ 0.05 and *P* ≤ 0.10.

Circulating IGF-1 concentrations tended to be higher in winter compared with summer, with a clear effect of day within each season observed (Table 3; *P* < 0.001). Insulin-like growth factor type 1 plays a significant role in regulating cell cycle and apoptosis, and it serves as a predictor of growth rate [81]. Increased circulating IGF-1 concentrations are related to increased feed intake and anabolism of proteins in muscles [82,83]. However, no increases in IGF-1 for more efficient animals were observed in this study (*P ≥* 0.56 in both seasons; Table 3), as detected previously in similar studies in high forage diets [84,85].

**Fig 7:**
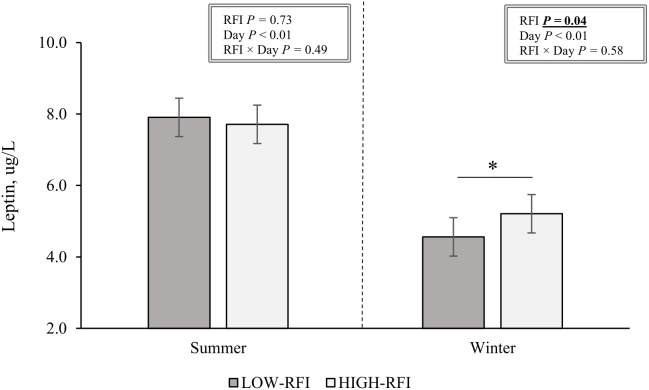
Plasma concentrations of leptin measured during the summer and winter (summer 2022 and winter 2023) in crossbred beef heifers previously classified as more (LOW-RFI) or less (HIGH-RFI) feed efficient. An effect of RFI was found with greater leptin concentrations in the HIGH-RFI compared with LOW-RFI heifers during the winter (*P* = 0.04; 5.2 vs. 4.6; SEM ± 0.122 ug/L, respectively). No effects of RFI were observed during the summer (*P* = 0.73). *Within RFI, means with an asterisk are different (*P* < 0.05). SEM: Standard error of the mean. RFI: Residual feed intake. HIGH-RFI: Less efficient individual tested for residual feed intake with a positive DMI. LOW-RFI: More efficient individual tested for residual feed intake with a negative DMI.

Our study found greater LEP concentrations in HIGH-RFI heifers when compared with LOW-RFI (*P* = 0.04; Fig 7; 5.2 vs. 4.6 ug/L, respectively) in the winter season. Leptin is a key indicator of energy reserves and body condition score, while also playing a crucial role in signaling the central nervous system and regulating feed intake [86]. By activating neuroendocrine pathways in the brain, LEP modulates metabolism and energy expenditure [87]. Previous studies have evaluated LEP concentrations in beef heifers and steers, finding that LEP was positive associated with the gain-to-feed ratio and negatively associated with RFI [88,89]. Our results, support these studies and suggest that more efficient cattle tend to have lower LEP concentrations. Furthermore, studies involving mice have indicated that LEP induces thermogenesis and helps maintain body temperature, partly through its influence on the thyroid hormone axis [90]. Additionally, LEP may support heat retention through vasoconstriction [91], potentially benefiting less feed-efficient animals during CS.

**Fig 8:**
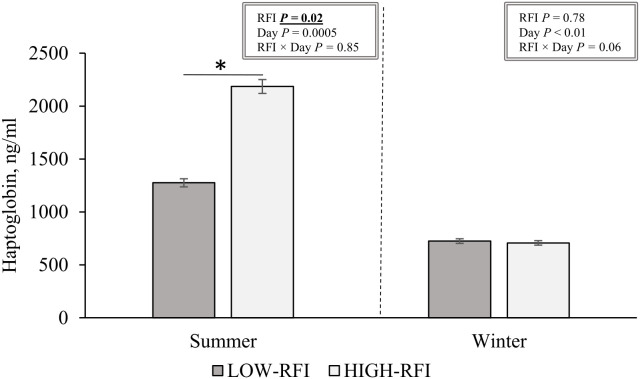
Plasma concentrations of haptoglobin measured during the summer and winter (summer 2022 and winter 2023) in crossbred beef heifers previously classified as more (LOW-RFI) or less (HIGH-RFI) feed efficient. During the summer, an effect of RFI was found with greater haptoglobin in the HIGH-RFI compared with the LOW-RFI in the summer season (*P* = 0.02; 2185 vs. 1274; SEM ± 25.61 ng/ml, respectively). In contrast, no significant differences were observed between groups during the winter season (*P* = 0.78). *Within RFI, means with an asterisk are different (*P* < 0.05). SEM: Standard error of the mean. RFI: Residual feed intake. HIGH-RFI: Less efficient individual tested for residual feed intake with a positive DMI. LOW-RFI: More efficient individual tested for residual feed intake with a negative DMI.

**Fig 9:**
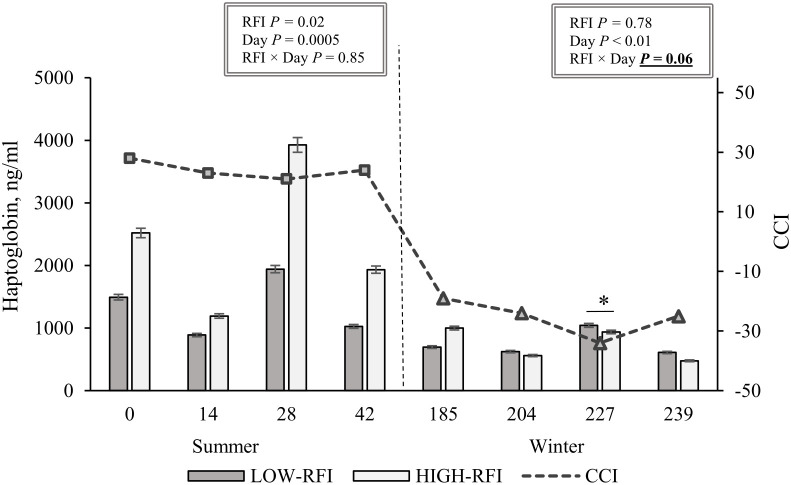
Plasma concentrations of haptoglobin (Hp) measured during the summer and winter (summer 2022 and winter 2023) in crossbred beef heifers previously classified as more (LOW-RFI) or less (HIGH-RFI) feed efficient. A tendency for an RFI × day interaction for Hp was detected, with greater concentrations in the LOW-RFI on the coldest sampling day (d 227; 1042 and 937; SEM ± 1.16 ng/ml) during the winter season (*P* = 0.06). *Within RFI, means with an asterisk tended to be different (*P* = 0.06). SEM: Standard error of the mean. RFI: Residual feed intake. HIGH-RFI: Less efficient individual tested for residual feed intake with a positive DMI. LOW-RFI: More efficient individual tested for residual feed intake with a negative DMI. CCI: Comprehensive Climate Index [36].

Haptoglobin is a protein released by the liver during an acute-phase response, a part of the body’s immediate reaction to inflammation processes, cell disruption, and stress [92]. Haptoglobin concentrations below 0.1 mg/mL have been previously reported in healthy cows [93]. Concentrations 100-fold under 0.1 mg/mL were found for HIGH vs. LOW-RFI heifers in the summer (*P* = 0.02; 2185 vs. 1274 ng/mL; Fig 8). A tendency for an RFI × day interaction was observed for Hp (*P* = 0.06; Fig 9). On day 227, with an extremely cold environment, Hp was greater in the LOW-RFI group. However, Hp concentrations in both groups and seasons were within the thresholds established for healthy animals.

**Fig 10:**
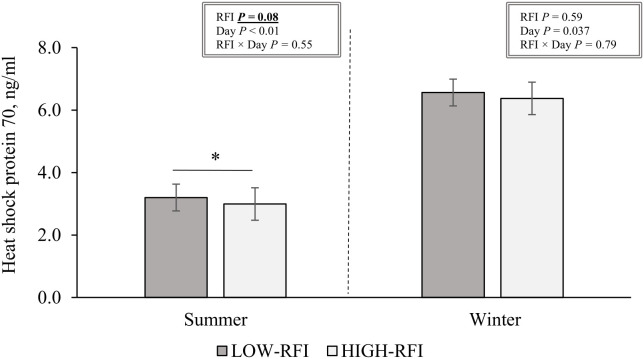
Plasma concentrations of heat shock protein 70 (HSP70) measured during the summer and winter (summer 2022 and winter 2023) in crossbred beef heifers previously classified as more (LOW-RFI) or less (HIGH-RFI) feed efficient. A tendency was found for the LOW-RFI group to produce more HSP70 compared with the HIGH-RFI in the summer (*P* = 0.08; 3.20 vs. 2.99; SEM ± 0.092 ng/ml). *Within RFI, means with an asterisk tended to be different (*P* = 0.08). SEM: Standard error of the mean. RFI: Residual feed intake. HIGH-RFI: Less efficient individual tested for residual feed intake with a positive DMI. LOW-RFI: More efficient individual tested for residual feed intake with a negative DMI.

Heat shock protein 70 is known to play a significant role during CS [94], and in the present study, concentrations of this protein were generally greater during winter than summer (6.48 vs. 3.10 ng/ml). In the summer, LOW-RFI heifers also tended to produce more HSP70 when compared with the HIGH-RFI cohort (*P =* 0.08; Fig 10; 3.20 vs. 2.99 ng/ml). However, this study did not find effects of RFI or RFI × day in the winter season (*P* ≥ 0.59, Fig 10). During both seasons, an effect of day was also observed (*P* < 0.037, Table 3). Concentrations of HSP70 were highest at day 42 shortly after exposure to mild climate risk (HS) on day 41, while it was at their lowest at day 28 (*P* < 0.001; Table 3). In another study, HSP70 concentrations were positively correlated with climate temperature, age, and fat deposition, but not with greater body temperature [95]. Additionally, lower concentrations of HSP70 have been observed in calves exposed to HS compared to those under cooling conditions [96]; that gene expression study is similar with the HIGH-RFI heifers tested in this study, suggesting that less efficient heifers, which produce less HSP70, are more susceptible to chronic HS than LOW-RFI heifers. Moreover, highlighting the enhanced biological function of LOW-RFI heifers during summer as greater HSP70 will inhibit the programmed cell death in LOW-RFI [97]. During the winter, HSP70 was found to be greater in heifers during the coldest sampling time (day 227; 7.23 ± 0.063 ng/ml), doubling the concentrations compared with our summer season. This corroborates with previous research indicating HSP70 is a suitable marker for CS detection [98].

**Fig 11:**
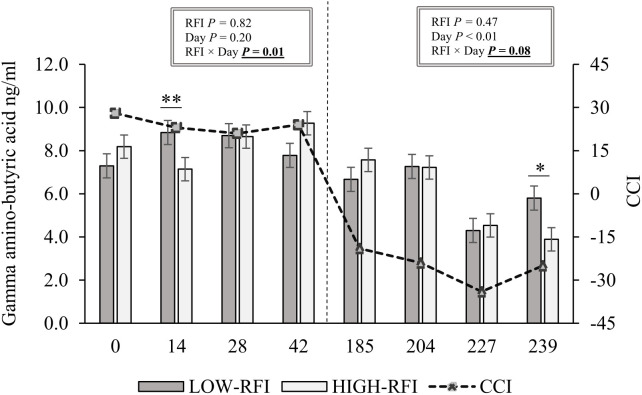
Plasma concentrations of gamma amino-butyric acid (GABA) measured during the summer and winter (summer 2022 and winter 2023) in crossbred beef heifers previously classified as more (LOW-RFI) or less (HIGH-RFI) feed efficient. An RFI × day interaction was observed for GABA with LOW-RFI having greater concentrations on day 14 (*P* = 0.01; 8.8 vs. 7.1; SEM ± 1.081 ng/ml). GABA levels tended to decrease when climate conditions reached severe cold on day 239 in HIGH-RFI heifers (*P* = 0.08; 3.9 vs. 5.8; SEM ± 1.117 ng/ml). * Within RFI, means with an asterisk tended to be different (*P* < 0.1). ** Within RFI, means with an asterisk are different (*P* < 0.05). SEM: Standard error of the mean. RFI: Residual feed intake. HIGH-RFI: Less efficient individual tested for residual feed intake with a positive DMI. LOW-RFI: More efficient individual tested for residual feed intake with a negative DMI.

An effect of day was detected in the current study for 5-HT in the summer and winter seasons (*P* < 0.001 for both seasons; Table 3), while no effects of RFI or RFI × day interactions occurred (*P* ≥ 0.14; Table 3). The 5-HT serves as an immunomodulatory biogenic amine, acting both as a neurotransmitter and a mediator in stress responses in heat-stressed dairy calves [96]. In the present study, 5-HT levels generally were greater during summer; however, they also decreased under severe and extreme cold conditions in winter. Serotonin is involved in thermoregulation processes [99], and a decreased concentration in the peripheral circulation of rodents following acute HS exposure has been reported [100]. Furthermore, chronic CS disrupts the modulatory neurotransmitter system, decreasing 5-HTlevels, as previously described [101]. In chronically stressed rats, the hypothalamus also shows a reduced number of receptors involved in 5-HT production [102]. Moreover, an RFI × day interaction (*P* = 0.01; Fig 11) was observed on GABA during summer. Gamma amino-butyric acid decreases body temperature and regulates stress responses [103,104] and is expected to decrease after hyperthermia in rabbits [105]. On day 14, the lowest concentration of GABA was observed for the HIGH-RFI group (*P =* 0.01; Fig 11). Overall, HIGH-RFI heifers had lower GABA concentrations than LOW-RFI heifers (*P* = 0.01; 7.1 vs. 8.8 ng/ml) during summer and tended to have lower concentrations of GABA in winter (*P* = 0.08; 3.9 vs. 5.8 ng/ml; Fig 11). Additionally, it has been reported that individuals with low plasma GABA levels are more vulnerable to stress-related disorders [106].

The genomic breed analysis revealed that the composition of Black Angus, Hereford, Gelbvieh, Charolais, Limousin, Brown Swiss, Galloway, Red Angus, Salers, Maine-Anjou, Shorthorn, Holstein, and Jersey was similar between the RFI groups (*P* > 0.13; 36 ± 9, 15 ± 3, 8 ± 4, 7 ± 3, 6 ± 3, 4 ± 2, 4 ± 2, 3 ± 2, 3 ± 2, 2 ± 2, 2 ± 2, 2 ± 2, 1 ± 1% for LOW-RFI and 34 ± 10, 15 ± 4, 10 ± 4, 8 ± 2, 6 ± 3, 3 ± 2, 5 ± 2, 4 ± 3, 2 ± 2, 1 ± 1, 3 ± 3, 3 ± 1, 1 ± 1% for HIGH-RFI). However, the proportion of Simmental tended to be greater in LOW-RFI compared to HIGH-RFI heifers (9 ± 3 and 7 ± 3%, respectively; *P* = 0.05). The gRHET mean by groups was 0.78 ± 0.052 for LOW-RFI and 0.79 ± 0.056 for HIGH-RFI. As expected, both groups showed moderate heterozygosity, reflecting the greater genetic diversity of crossbred cattle [107]. Genomic heterozygosity exhibits a linear relationship with heterosis, overall fitness, resilience, and lifetime productivity in cattle [108]. In this study, no significant differences in gRHET averages were observed between the LOW-RFI and HIGH-RFI groups (0.78 vs. 0.79 SEM ± 0.057, respectively *P* = 0.69), and the low standard deviation of the groups represents a negligible variation of gRHET values within RFI groups. Therefore, it is unlikely that differences in weather stress responses were due to genomic heterozygosity. Thus, crossbred beef heifers with greater RFI classification (reduced feed efficiency) may be more susceptible to the negative effects of weather stress.

## Conclusion

Our study highlights the significant climate challenges faced by beef cattle in Western Canada, ranging from non-stressful to severe conditions during the summer and from non-stressful to extremely stressful conditions during the winter season. During summer, HIGH-RFI (less feed-efficient) heifers exhibited higher RT, lower GABA concentrations, and tended to have lower fT3, indicating greater susceptibility to HS, whereas LOW-RFI heifers maintained more stable body temperatures and tended to have higher HSP70 expression, suggesting superior thermoregulatory capacity. In winter, LOW-RFI heifers maintained higher RT and tended to have higher GABA, which might indicate enhanced resilience to CS. Leptin concentrations were higher in HIGH-RFI heifers during winter, potentially supporting heat retention under cold conditions, while BUN, NEFA, and BHBA remained generally stable across RFI groups, with transient NEFA elevations under extreme cold indicating short-term negative energy balance. Importantly, no differences in growth performance were observed despite numerous changes to metabolic indicators. Genomic analysis confirmed similar breed composition and heterozygosity between groups, suggesting that observed physiological differences are not attributable to genetic diversity. Overall, these findings suggest that feed-efficient (LOW-RFI) heifers exhibit enhanced physiological and metabolic resilience to both heat and CS, while less efficient cattle are more vulnerable to climatic challenges. This highlights the potential of RFI as a selection criterion for resilient, climate-adapted cattle, contributing to improved beef production and animal welfare in challenging weather conditions.
